# Unveiling the Structural Properties, Optical Behavior, and Thermoelectric Performance of 2D CsSn_2_Br_5_ Halide Obtained by Mechanochemistry

**DOI:** 10.1021/acs.inorgchem.4c01861

**Published:** 2024-06-26

**Authors:** Carlos Alberto López, Carmen Abia, Javier Gainza, João Elias Rodrigues, Brenda Martinelli, Federico Serrano-Sánchez, Romualdo Santos Silva, Mateus M. Ferrer, Oscar J. Dura, José Luis Martínez, María Teresa Fernández-Díaz, José Antonio Alonso

**Affiliations:** † Instituto de Ciencia de Materiales de Madrid, CSIC, Cantoblanco, 28049 Madrid, Spain; ‡ INTEQUI, (UNSL-CONICET) and Facultad de Química, Bioquímica y Farmacia, UNSL, Almirante Brown 1455, 5700 San Luis, Argentina; § Institut Laue Langevin, 38042 Grenoble, Cedex, France; ∥ European Synchrotron Radiation Facility (ESRF), 71 Avenue des Martyrs, 38000 Grenoble, France; ⊥ CELLS−ALBA Synchrotron Light Facility, Cerdanyola del Valles, Barcelona E-08290, Spain; # CCAF, PPGCEM/CDTec, Federal University of Pelotas, 96010-610 Pelotas, Rio Grande do Sul, Brazil; ∇ Departamento de Física Aplicada, Universidad de Castilla-La Mancha, Ciudad Real E-13071, Spain

## Abstract

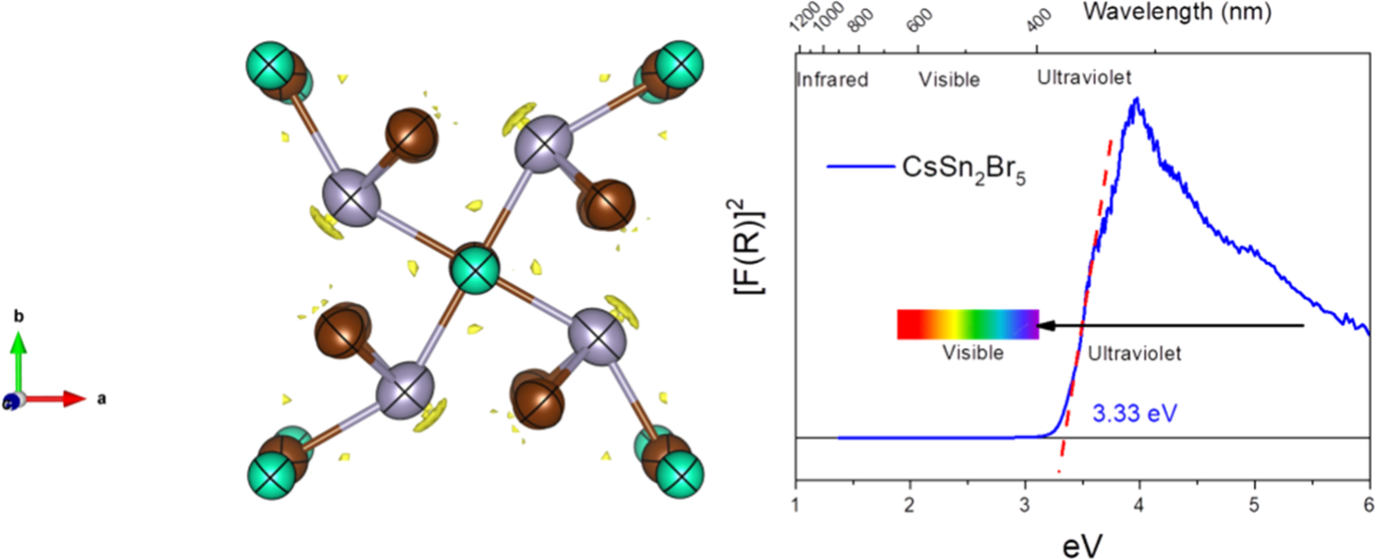

Metal halide perovskites with a two-dimensional structure are utilized in photovoltaics and optoelectronics. High-crystallinity CsSn_2_Br_5_ specimens have been synthesized via ball milling. Differential scanning calorimetry curves show melting at 553 K (endothermic) and recrystallization at 516 K (exothermic). Structural analysis using synchrotron X-ray diffraction data, collected from 100 to 373 K, allows for the determination of Debye model parameters. This analysis provides insights into the relative Cs–Br and Sn–Br chemical bonds within the tetragonal structure (space group: *I*4/*mcm*), which remains stable throughout the temperature range studied. Combined with neutron data, X–N techniques permit the identification of the Sn^2+^ lone electron pair (5s^2^) in the two-dimensional framework, occupying empty space opposite to the four Sn–Br bonds of the pyramidal [SnBr_4_] coordination polyhedra. Additionally, diffuse reflectance UV–vis spectroscopy unveils an indirect optical gap of approximately ∼3.3 eV, aligning with the calculated value from the *B3LYP*-DFT method (∼3.2 eV). The material exhibits a positive Seebeck coefficient as high as 6.5 × 10^4^ μV K^–1^ at 350 K, which evolves down to negative values of −3.0 × 10^3^ μV K^–1^ at 550 K, surpassing values reported for other halide perovskites. Notably, the thermal conductivity remains exceptionally low, between 0.32 and 0.25 W m^–1^ K^–1^.

## Abstract 2

2D CsSn_2_Br_5_, prepared by mechanochemistry, is studied from SXRD and NPD data in the 100−373 K interval. The Sn^2+^ lone electron pair is identified by X–N techniques. UV−vis spectroscopy unveils an optical gap of ∼3.3 eV, matching the calculated value from the *B3LYP*-DFT method (∼3.2 eV). The material demonstrates a remarkable thermoelectric performance comparable to those of other tin and lead halides, including a significant positive Seebeck coefficient and low thermal conductivity.

## 1 Introduction

Since the pioneering work by Grätzel,^1,2^ hybrid halide organic–inorganic perovskites constitute the new paradigm for solar energy conversion. These materials have gained significant popularity in recent years for their potential in photovoltaic applications, achieving promising power conversion efficiencies exceeding 23%.^3,4^

The all-inorganic version of these perovskite halides also stands out as excellent materials for solar cell applications, boasting intriguing optical properties, such as band-gap tuning and high quantum efficiency. Among these, cubic CsPbI_3_, which has a band gap of 1.73 eV,^5^ demonstrates high fluorescence quantum yield and increased resistance to degradation in ambient atmosphere and humidity. However, a significant challenge impeding its commercialization is stabilizing the cubic α-CsPbI_3_ phase at room temperature (RT) in ambient atmosphere and humidity, as this cubic phase tends to transition to an undesired orthorhombic (δ) symmetry.^6^

Addressing this challenge, the substitution, whether complete or partial, of I with Br proves to be a viable solution, as seen in the case of orthorhombic CsPbBr_3_ perovskite.^7−11^ These chemical modifications induce changes in the optoelectronic properties, resulting in a high and consistent photoresponse and stable performance under ambient conditions. As a drawback, the I-to-Br substitution increases the band gap of the CsPbBr_3_ perovskite to 2.3 eV, limiting its applicability in solar cell devices, although it remains suitable for various optoelectrical systems.^12^

Recent efforts to enhance photoefficiencies in these halide perovskites involve diverse strategies, such as structural ordering and alternative topologies for octahedral arrangements, as demonstrated by CsPb_2_Br_5,_^13−15^ belonging to the APb_2_X_5_ family (where A = K, Rb, Cs and X = Cl, Br). These halides deviate from the classical three-dimensional (3D) network of corner-sharing [PbX_6_] units, displaying a two-dimensional (2D) topology with an intriguing layered structure.^16^ Another noteworthy member of this layered lead-containing halide family is RbPb_2_Br_5_, identified as a promising material with low-phonon energy for tunable infrared laser sources in the mid- and long-wavelength ranges, suitable for applications like remote sensing.^17,18^ Despite its potential, the presence of toxic Pb poses a significant obstacle to its practical use and commercialization.

To overcome this limitation, extensive efforts have been directed toward developing Pb-free options, exemplified by cesium di-tin pentabromide (CsSn_2_Br_5_) belonging to the 2D family of layered tin-containing halides. While recent estimates have shed light on its optical and dielectric properties,^19−21^ a comprehensive exploration of its crystallographic structure and transport properties, including electronic conductivity, Seebeck coefficient, and thermal transport, is notably absent. Furthermore, layered materials have demonstrated intriguing thermoelectric properties, as observed in compounds like NaCo_2_O_4_,^22^ Bi_2_Te_3_,^23,24^ silicene^25^ and phosphorene,^26^ and chalcogenides like SnSe, achieving a figure of merit for thermoelectric materials (*ZT* = *S*^2^σ*T*/κ) of ∼2.8 due to an exceptionally low thermal conductivity of ∼0.2 W/m K.^27−30^

Noteworthy thermoelectric performances have also been observed in polycrystalline bulk forms of hybrid and all inorganic halide perovskites,^31−35^ such as CsSnBr_3–*x*_I_*x*,_^31^ proposed as potential thermoelectric materials. Despite a modest figure of merit (∼0.15), their exceptionally low thermal conductivity (0.32 W/m K for CsSnBrI_2_) positions them as candidates for promising thermoelectric applications.^32^ Extremely low thermal conductivities have been described in polycrystalline halide perovskites, including 0.3–0.5 W/m K reported for the MAPI_3_ (CH_3_NH_3_PbI_3_) perovskite,^33^ 0.43 and 0.33 W/m K for CsPbBr_3_ and CsPb_2_Br_5,_^34^ respectively, and 0.74 W/m K reported for the CsSnI_3_ compound.^35^

This study describes the successful synthesis of a polycrystalline sample of CsSn_2_Br_5_ using a solvent-free mechanochemical method with a planetary ball mill. We conducted a neutron powder diffraction (NPD) experiment to examine the crystallographic structure across a broad temperature range (295–550 K), complemented by synchrotron X-ray diffraction (SXRD) data from 100 to 373 K. The specimen exhibits a tetragonal symmetry described by the *I*4/*mcm* (#140) space group, with no observed phase transitions below the melting point. The thermal variation of the atomic mean-square displacement factors yields the Debye temperatures, enabling the estimation of the bonding covalence in CsSn_2_Br_5_. UV–vis spectra and ab initio calculations confirm an optical gap of 3.3 eV (calculated indirect band gap of 3.2 eV). Additional characterization, including thermal analysis and transport properties, reveals a substantial Seebeck coefficient of ∼2 × 10^4^ μV/K at 400 K.

## 2 Experimental Methods

### 2.1 Mechanochemical Synthesis

Mechanochemical synthesis was employed to produce CsSn_2_Br_5_ in a polycrystalline powder form. The synthesis involved a planetary ball mill and stoichiometric amounts of SnBr_2_ and CsBr (1 g in total mass). This mixture, along with 20 zirconia balls (5 mm diameter), was combined in a N_2_-filled glovebox and subjected to a 3 h reaction at 450 rpm within a sealed zirconia-lined jar under a N_2_ atmosphere, using a Retsch PM100 mill.

### 2.2 Structural Characterization and Analysis

For structural characterization and analysis, a Bruker D5 diffractometer with Cu Kα radiation (λ = 1.5418 Å) was used to collect a laboratory XRD pattern at room temperature. The thermal evolution of the crystallographic structure was studied by SXRD, at room temperature (30 °C) and 100 °C; SXRD patterns were collected in high angular resolution mode (so-called MAD setup) on the MSPD diffractometer in CELLS-ALBA synchrotron in Barcelona (Spain), selecting an incident beam with 38 keV energy (λ = 0.325760 Å).^36^ The sample was contained in a 0.5 mm diameter quartz capillary that was rotating during the data acquisition. Additionally, low-temperature patterns between 100 and 200 K were collected at the ID22 diffractometer^37^ in the ESRF synchrotron (Grenoble) with λ = 0.35434 Å (35 keV). NPD patterns were collected at RT at the D1B instrument, a medium-flux diffractometer in the ILL reactor (Grenoble) with a wavelength of 1.280 Å. The obtained SXRD and NPD data underwent the Rietveld analysis using the *FullProf* software.^38,39^ The refined parameters were the following: zero-point error, background coefficients, scale factor, asymmetry factors, lattice parameters (*a*, *c*), atomic fractional coordinates (*x*, *y*, *z*), and isotropic thermal displacements (*U*_iso_). The neutron scattering lengths for Cs, Sn, and Br atoms are 5.420, 6.225, and 6.795 fm, respectively.

### 2.3 Thermal and Morphological Characterizations

The thermal characterizations involved DSC measurements in a Mettler TA3000 system in the range of 130–520 K. The heating and cooling rates were 10 K min^–1^, using about 70 mg of sample. Thermogravimetric curves were measured in a TG50 microbalance. 50 mg of sample was treated at a heating rate of 10 °C min^–1^ in a nitrogen flow, from 300 to 673 K. The morphological studies were performed by means of field-effect scanning electron microscopy (FE-SEM), with the images being recorded using an FEI Nova microscope, with an acceleration potential of 5 kV, coupled to an energy-dispersive X-ray spectrometer (EDX), working with an acceleration voltage of 18 kV and 60 s of acquisition time.

### 2.4 Optical Properties

Optical diffuse reflectance spectrum measurement used a Varian Cary 5000 UV–vis spectrophotometer. The absorption capacity of CsSn_2_Br_5_ was investigated by diffuse reflectance UV–vis spectroscopy. The UV–vis spectrum was used to calculate the optical absorption coefficient (α), since it is related to the Kubelka–Munk function [*F*(*R*) ∝ α = (1 – *R*)^2^/2*R*, *R* being the reflectance versus wavelength in eV].

### 2.5 Thermoelectric Characterization

For thermoelectric characterization, the synthesized powder was cold-pressed into a pellet, achieving around ∼93% compared to the theoretical crystallographic density. The Seebeck coefficient was derived by measuring simultaneously drop voltages across the sample and a constantan reference wire with an electrometer (Keithley 6517B) and nanovoltmeter (Keithley 2182A) under vacuum (10^–3^ mbar). Electrical resistivity was measured using an Agilent E4980A LCR meter. The total thermal conductivity was determined through the laser-flash technique in a Linseis LFA 1000 equipment, calculated from thermal diffusivity, specific heat, and sample density. For a comprehensive overview and application examples of our custom thermoelectric characterization system, please refer to the cited reference for further details.^40^

## 3 Computational Methods

To support the understanding of electronic transitions and the chemical environment, theoretical models were created based on density functional theory. The models were tested using the *CRYSTAL17*(41) package with the *B3LYP* functional.^42^ The atomic bases used in the calculations were all triple-zeta valence with polarization quality (POB-TZVP) developed by Bredow et al.^43^ The thresholds of the Coulomb and exchange series are controlled according to the parameters of superposition and penetration for Coulomb integrals, superposition for HF exchange integrals, and pseudosuperposition, defined, respectively, as 8, 8, 8, 8, and 16. The long-range electron correlation was considered based on the Grimme D3 semiempirical correction.^44^ The shrinkage factors (Pack–Monkhorst and Gilat network) were 6 and 6. The gradient component and nuclear shift of the structure optimization were adjusted with a tolerance on their root-mean-square value of 0.0003 and 0.0012 au, respectively. The topological analysis of the critical points of chemical bonds was carried out according to the “quantum theory: atoms in molecules” (QTAIM) with the *CRYSPLOT* program, part of the *CRYSTAL17* package.

## 4 Results and Discussion

### 4.1 Initial Characterization

**Figure 1:**
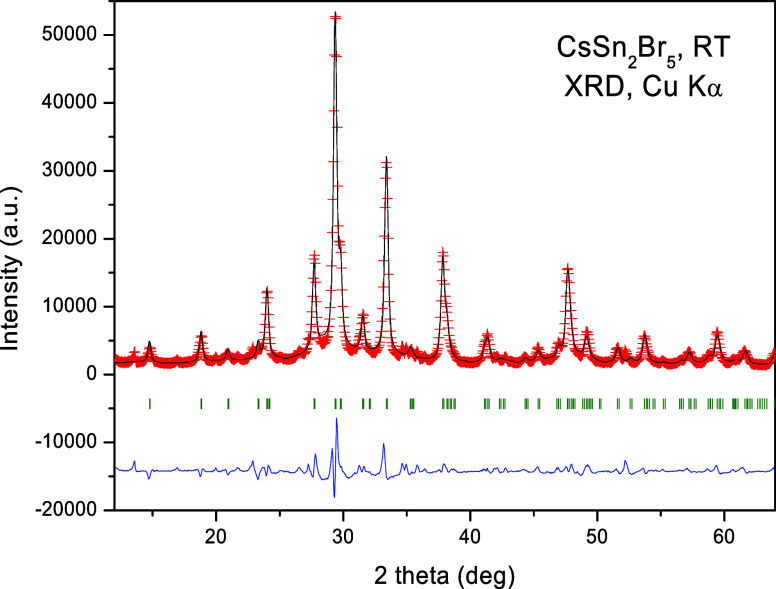
Le Bail fit for the mechanochemically prepared CsSn_2_Br_5_ at room temperature from laboratory XRD data from Cu Kα radiation.

CsSn_2_Br_5_ was obtained as a pale-cream microcrystalline powder. The sample was initially identified from laboratory X-ray diffraction data at RT, as illustrated in a Le Bail fit displayed in Figure 1. The diffraction pattern is in agreement with the tetragonal crystal structure reported more than 20 years ago by Abrahams et al. from single-crystal laboratory X-ray diffraction.^45^

### 4.2 Thermogravimetric Analysis

**Figure 2:**
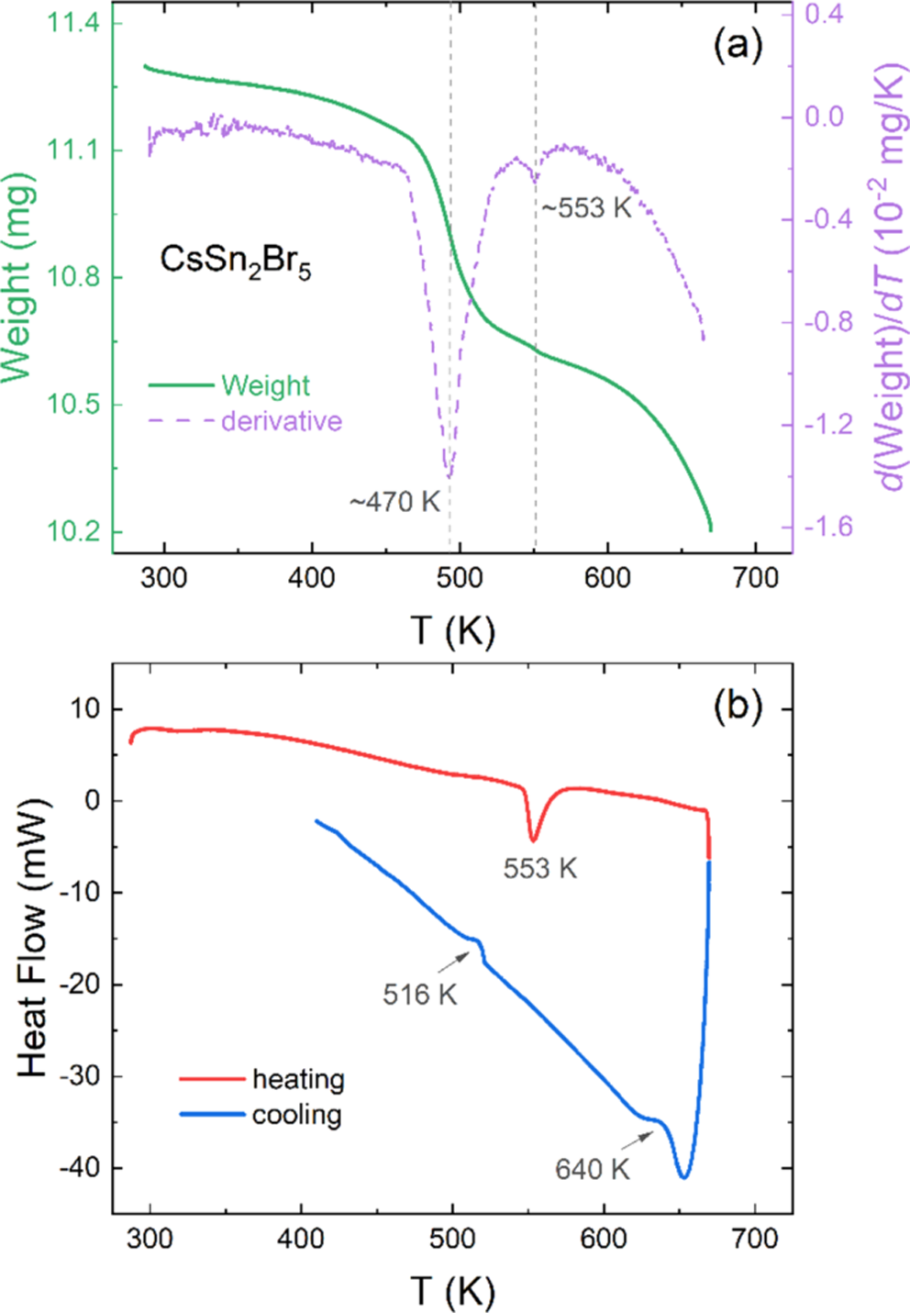
Thermogravimetric curve and its respective derivative [d(weight)/d*T*] showing weight loss evolution of the CsSn_2_Br_5_ sample (a). DSC curve emphasizing the endothermic and exothermic peaks in the respective heating and cooling runs (b).

The thermogravimetric analysis of the synthesized CsSn_2_Br_5_ is shown in Figure 2a. Above 400 K, the curve exhibits a significant weight loss of 5.2% centered at 470 K, followed by a smaller loss of 0.6% centered at 553 K, as determined through the derivative curves d(weight)/d*T*, which are attributed to the excess bromine elimination. In Figure 2b, one cycle of the DSC curve is displayed, with their respective cooling and heating runs. Reversible peaks with non-negligible hysteresis are observed, with an endothermic peak at 553 K (heating run) and exothermic peaks at 516 and 640 K (cooling run), which likely correspond to the melting and structural recrystallization of the CsSn_2_Br_5_ sample.

### 4.3 SEM

**Figure 3:**
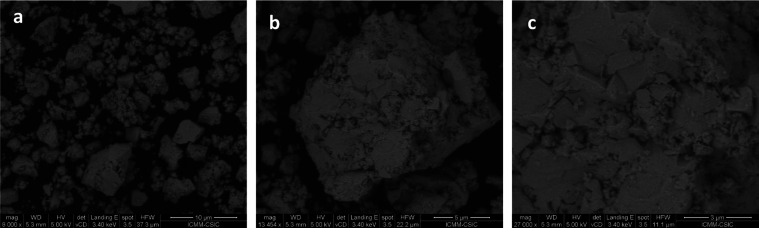
FE-SEM images with 8000× (a), 13 454× (b), and 27 000× (c) magnifications.

FE-SEM images are displayed in Figure 3, providing insights into the microstructure of the product synthesized using ball milling. At low magnification (8000×), clusters of particles with irregular shapes and different sizes are visible (see in Figure 3a). However, at higher magnification (13 454× and 27 000×), Figure 3b,3c reveals that these clusters are indeed composed of compact microparticles with sharp edges, typically measuring between 0.5 and 1 μm. These particles are grown during the ball milling process. The semiquantitative EDX analysis coupled with the FE-SEM images indicates an atomic composition close to 1:2:5 for the Cs/Sn/Br ratio. A characteristic EDX spectrum is presented in Figure S1 in the Supporting Information, and more SEM images are displayed in Figure S2.

### 4.4 Crystal Structure Analysis

As mentioned above, an initial structural characterization was carried out from laboratory X-ray diffraction data at room temperature (RT). The CsSn_2_Br_5_ sample displayed a tetragonal symmetry indexable within the *I*4/*mcm* space group (#140).^45^

A more in-depth structural analysis was conducted using both synchrotron X-ray and neutron diffraction data. Both patterns at RT confirmed the tetragonal symmetry (S.G: *I*4/*mcm*). In this space group, Cs^+^ and Sn^2+^ ions occupy 4*a* (0,0,1/4) and 8*h* (*x*, *x* + 1/2,0) Wyckoff sites, respectively. There are two types of bromine atoms, Br1 and Br2, located at 4*c* (0,0,0) and 16*l* (*x*,*y*,*z*) positions, respectively.

**Figure 4:**
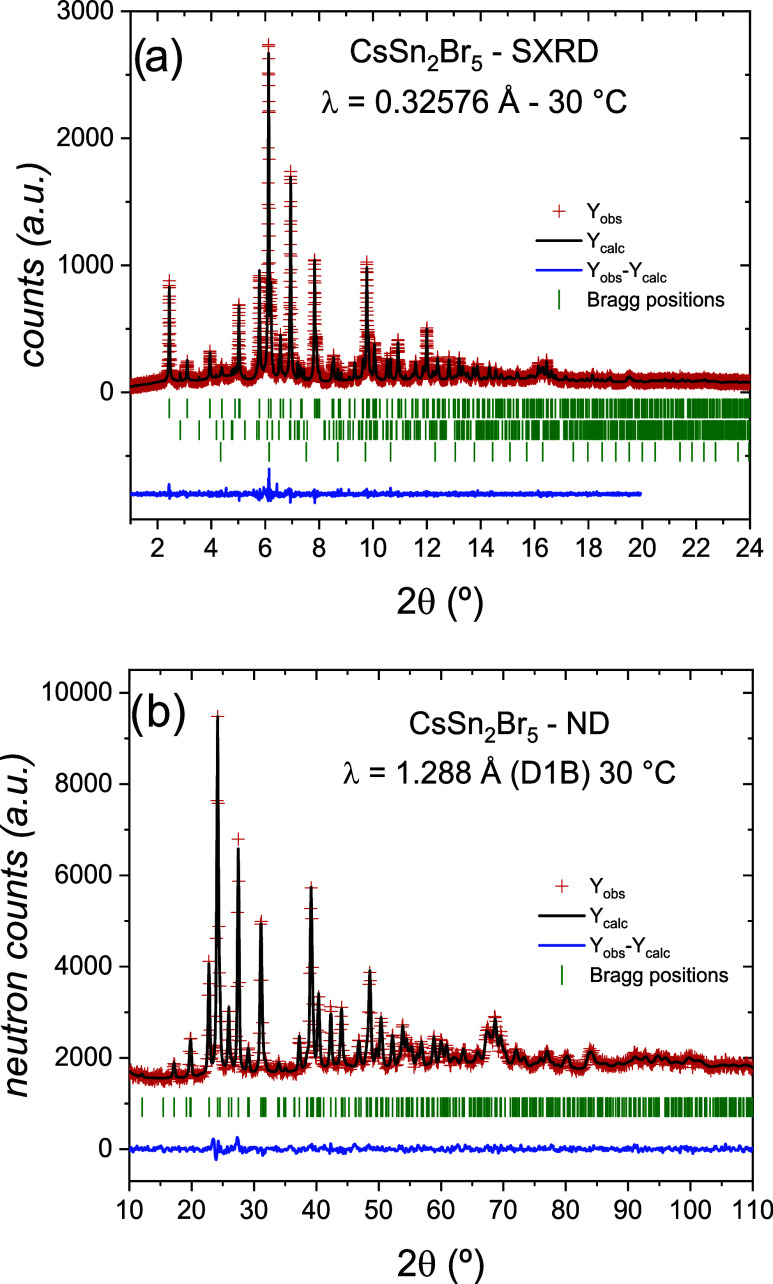
Rietveld plots for CsSn_2_Br_5_ at room temperature, where the red crosses represent the observed profile; the full black line is the calculated profile, and the difference is the blue line below. The Bragg positions are displayed as green vertical bars. (a) SXRD and (b) NPD data.

**Figure 5:**
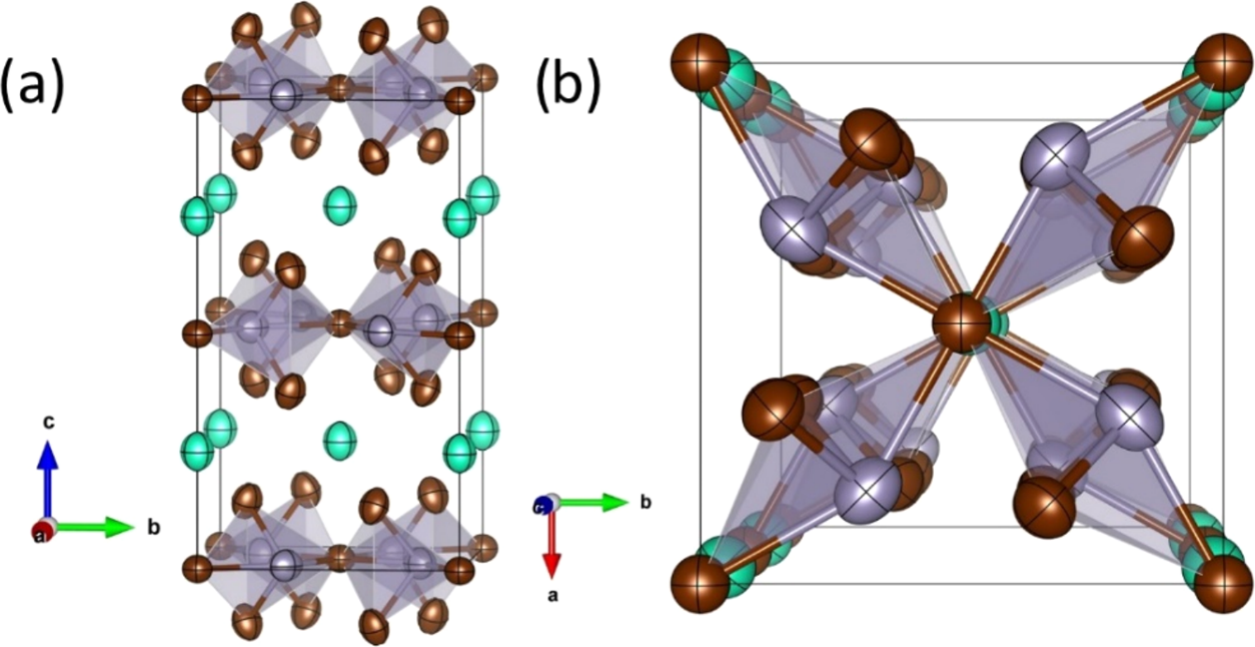
Views along the *a*-axis (a) and *b*-axis (b) of the layered crystal structure of CsSn_2_Br_5_; the [SnBr_4_] polyhedra establish layers within the *ab* plane; Cs atoms are intercalated in between; the distribution of the four Sn–Br bonds in square pyramids is driven by the 5s^2^ lone pair repulsion. The green, gray, and brown atomic representations denote cesium (Cs), lead (Pb), and bromide (Br) atoms, respectively.

The corresponding Rietveld refinement at room temperature obtained from SXRD data is presented in Figure 4a. The refined unit cell parameters at RT are *a* = 8.5036(3) Å, *c* = 15.2992(6) Å, and *V* = 1106.30(7) Å^3^. In Table 1, the main crystallographic parameters obtained from SXRD are listed. The NPD data were also fitted, and the Rietveld refinement is plotted in Figure 4b. In Table 2, the main crystallographic parameters obtained from NPD are included. In this case, a combined refinement from both types of data, was not pertinent since we aimed at performing an X–N study, taking advantage of the peculiarities of both radiations, as described below. Two views of the crystal structure of CsSn_2_Br_5_ at room temperature from NPD data are represented in Figure 5. The structure consists of layers of corner-sharing [SnBr_4_] polyhedra (through Br1 atoms), constituting square pyramids with Sn in the apex, connected by Cs^+^ ions. The stereochemical effect of the 5s^2^ lone electron pairs of Sn^2+^ is responsible for this asymmetrical configuration, as it was recently demonstrated in its Rb^+^ counterpart, RbSn_2_Br_5_.^46^

**Table 1:** Crystallographic Parameters for the CsSn_2_Br_5_ Phase in the Tetragonal System (*I*4/*mcm*) from SXRD Data at Room Temperature^a^,^b^

	*x*	*y*	*z*	*U*_iso_ (Å^2^)		*f*_occ_
Cs	0	0	0.25	0.042(2)		1
Sn	0.1796(2)	0.6796(2)	0	0.037(2)		1
Br1	0	0	0	0.028(3)		1
Br2	0.6594(2)	0.1594(2)	0.1285(1)	0.036(2)		1
Atomic Displacement Parameters (Å^2^)						
	*U*^11^	*U*^22^	*U*^33^	*U*^12^	*U*^13^	*U*^23^
Cs	0.039(2)	0.038(2)	0.050(3)	0	0	0
Sn	0.039(1)	0.039(1)	0.033(2)	–0.009(2)	0	0
Br1	0.029(2)	0.029(2)	0.025(4)	0	0	0
**Br2**	0.032(1)	0.032(1)	0.044(2)	–0.003(1)	0.006(1)	0.006(1)

a *a* = 8.5036(3) Å, *c* = 15.2992(6) Å, and *V* = 1106.30(7) Å^3^.

b *R*_p_ = 5.8%, *R*_wp_ = 7.3%, χ^2^ = 1.14, *R*_Bragg_ = 3.15%.

**Table 2:** Crystallographic Parameters for the CsSn_2_Br_5_ Phase in the Tetragonal System (*I*4/*mcm*) from NPD Data at RT^a^,^b^

	*x*	*y*	*z*	*U*_iso_ (Å^2^)		*f*_occ_
Cs	0	0	0.25	0.037(8)		1
Sn	0.1807(5)	0.6807(5)	0	0.035(3)		1
Br1	0	0	0	0.018(5)		1
Br2	0.6595(3)	0.1595(3)	0.1286(2)	0.031(2)		1
Atomic Displacement Parameters (Å^2^)						
	*U*^11^	*U*^22^	*U*^33^	*U*^12^	*U*^13^	*U*^23^
Cs	0.035(6)	0.035(6)	0.042(1)	0	0	0
Sn	0.034(3)	0.034(3)	0.038(4)	–0.004(4)	0	0
Br1	0.020(4)	0.020(4)	0.013(6)	0	0	0
Br2	0.031(2)	0.031(2)	0.032(3)	0.000(2)	0.007(2)	0.007(2)

a *a* = 8.502(1) Å, *c* = 15.305(2) Å, and *V* = 1106.2(3) Å^3^.

b *R*_p_ = 2.0%, *R*_wp_ = 2.5%, χ^2^ = 1.35, *R*_Bragg_ = 3.25%.

**Figure 6:**
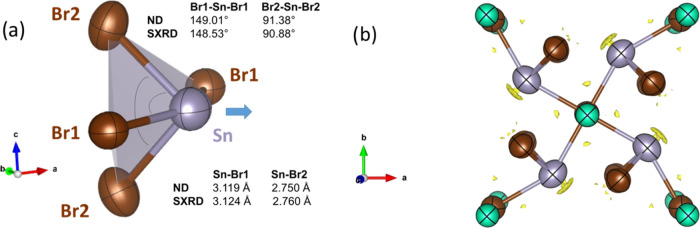
(a) [SnBr_4_] polyhedron and differences in distances and angles obtained from SXRD and NPD. (b). Distribution of the residual electron density, from X–N techniques, superposed with the [SnBr_4_] pyramidal structure.

From a comparative analysis of the obtained crystal structures, it is possible to identify some conspicuous differences in the Sn–Br polyhedron. The Sn–Br distances refined from NPD are shorter than those resolved from SXRD. Furthermore, the Br–Sn–Br angles in the structure obtained from NPD are greater than those obtained from SXRD. These concurrent changes are associated with a displacement in the Sn atom in the structure obtained from SXRD with respect to NPD, as illustrated in Figure 6a. Considering the differences between neutron and X-ray diffraction phenomena, this crystallographic distinction is attributed to a displacement in the electron cloud as a consequence of the lone pair effect in Sn^2+^ (5s^2^), as it was suggested by the [SnBr_4_] geometry. To confirm this, the so-called X–N method was employed. In this method, neutron data establish the positions of the nuclei, which are used to perform difference Fourier syntheses from SXRD data, involving the information on the electronic clouds. The result contains information on the electron density distribution in the crystal. Subsequently, difference Fourier maps were generated, and asymmetric densities were indeed found around Sn^2+^ cations. In Figure 6b, the difference electron density obtained is represented, where a strong density can be observed near Sn^2+^ ions and opposite of Sn–Br bonds. The position and intensity of these densities confirm the presence of the 5*s*^2^ lone electron pair of Sn^2+^, the stereochemical effect of which on the distribution of the chemical bonds was already commented. Consequently, the distorted coordination polyhedra of these cations on pseudosquare pyramids are driven by the lone electron pairs of Sn^2+^, which tend to occupy the empty space of the crystal structure, as shown in Figure 5a,b.

### 4.5 Lattice Dynamics

The study of temperature-dependent SXRD data allowed us to determine the thermal evolution of the crystal structure. The synchrotron pattern at 373 K (collected at ALBA) does not show any structural changes. Additional SXRD patterns (collected at ESRF) in the 100–200 K temperature range were all indexed in the tetragonal system. The thermal evolution of volume and unit cell parameters is plotted in Figure S3 of the Supporting Information.

**Figure 7:**
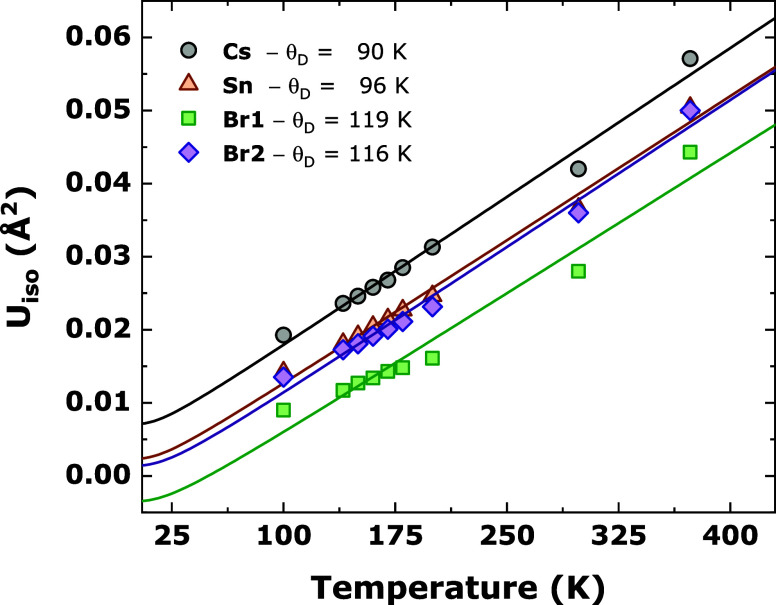
Thermal evolution of the isotropic displacement parameters (*U*_iso_) for the different atoms within the CsSn_2_Br_5_ crystal structure.

The thermal variation of the mean-square displacements (MSDs) of the atomic species Cs, Sn, and Br within the system CsSn_2_Br_5_ was analyzed by the Debye model.^47,48^ This model employs the isotropic displacement parameters (*U*_iso_, in units of Å^2^) as derived from the SXRD diffraction in the 100–373 K, as follows
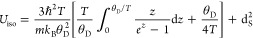
1where *m* stands for the atomic mass, *k*_B_ is the Boltzmann constant, ℏ is the reduced Planck constant, and *T* is the absolute temperature. After the nonlinear regression, the Debye temperature (θ_D_) and quadratic static displacement (*d*_S_^2^) parameters are obtained. In Figure 7, the fittings of the *U*_iso_ to the Debye model for CsSn_2_Br_5_ are represented. A reasonable agreement between experimental and fitted data allowed us to estimate the individual Debye temperature for all of the crystallographic sites of Cs, Sn, Br1, and Br2. The estimated θ_D_ values for these sites are 90, 96, 119, and 116 K, respectively. From the Debye temperature, the bond stiffness can be estimated considering the one-particle potential model for providing the approximated force constant . In fact, all of the obtained force constants are quite similar, as follows 0.63 (Cs), 0.65 (Sn), 0.67 (Br1), and 0.64 (Br2) eV/Å^2^, but slightly higher for Sn when compared to Cs, denoting a more covalent character for Sn–Br bonds. Similar tendencies were already reported by our group for other halide materials, such as Cs_4_PbBr_6–*x*_I_*x*_,^49^ RbSn_2_Br_5_,^46^ and RbPb_2_Br_5_,^50^ where (Cs,Rb)–(Br,I) bonds exhibited an ionic character compared to the more covalent (Pb,Sn)–(Br,I) bonds.

**Figure 8:**
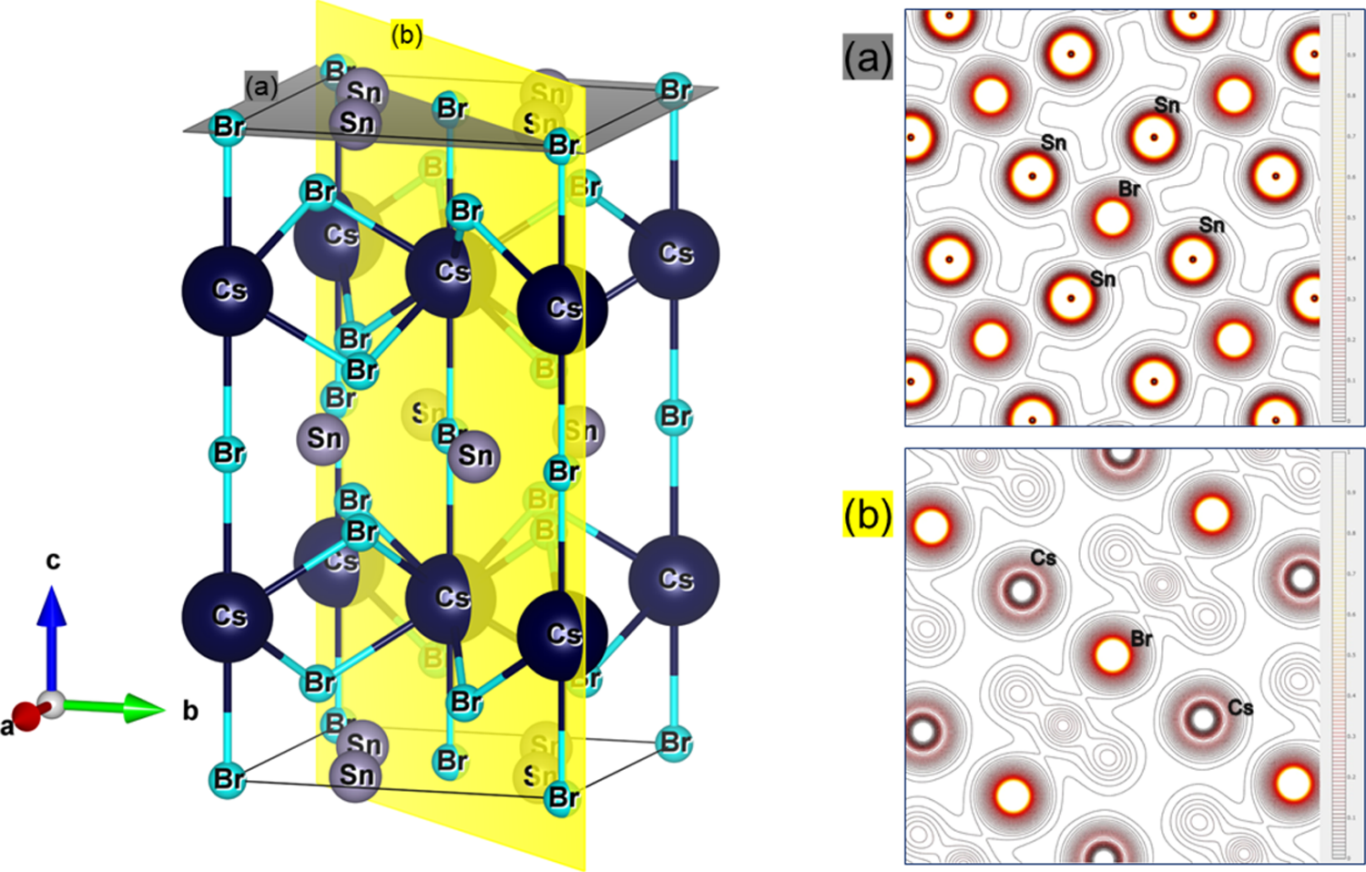
CsSn_2_Br_5_ unit cell and the electron density map of [001] (a) and [11̅0] (b) planes. Those planes were chosen to provide a better visualization of the topochemical isolines between Cs–Br and Sn–Br bonds.

To closely evaluate the nature of Cs–Br and Sn–Br pair bonds, the critical point parameters for each type of CsSn_2_Br_5_ bonds were calculated and are listed in Table 3. The types of bonds present in the structure can be estimated from the topological parameters. All of the bonds have relatively small ρ values, accompanied by positive ∇^2^ρ values, predominantly indicating an ionic character.^51,52^ However, there are significant differences between the Cs–Br and Sn–Br bonds. Initially, a more concentrated electron density is observed along the Sn–Br pair bond than in the Cs–Br pair. Opposite magnitudes are observed for the *H* parameters, which are positive for Cs–Br bonds and negative for Sn–Br bonds, and the |*v*|/*G* parameters are less than 1 for Cs–Br bonds, while for Sn–Br bonds, they vary between 1 and 2. This numerical behavior of Sn–Br bonds suggests a transient behavior, indicating the presence of a non-negligible covalent character and in agreement with the experimental results. The electron density maps of the [001] and [11̅0] planes can be seen in Figure 8. The planes were chosen to highlight the Cs–Br and Sn–Br bonds. The maps exhibit the shared isolines between the Sn–Br bonds (see Figure 8a) and the isolated isolines of the Cs ions (see Figure 8b).

**Table 3:** Critical Point Parameters in the Topological Analysis of CsSn_2_Br_5_: Bond Length (Å), Electron Density (ρ), Laplacian of Electron Density (∇^2^ρ), Virial Field Density (*v*), Lagrangian Kinetic Energy Density (*G*), and the Total Energy (*H*) of the Bonds

bond	*d* (Å)	ρ (x10^–3^)	∇^2^ ρ (x10^–2^)	*G* (x10^–3^)	*v* (x10^–3^)	*H* (x10^–3^)	*|v|*/*G*
Cs–Br_(1)_	3.70	9.00	2.54	5.55	–4.75	0.80	0.86
Cs–Br_(2)_	3.62	10.46	2.85	6.33	–5.54	0.79	0.87
Sn–Br_(1)_	3.07	25.13	4.21	12.14	–13.73	–1.60	1.13
Sn–Br_(2)_	2.76	44.86	7.09	24.45	–31.16	–6.80	1.28

### 4.6 Optical Properties

**Figure 9:**
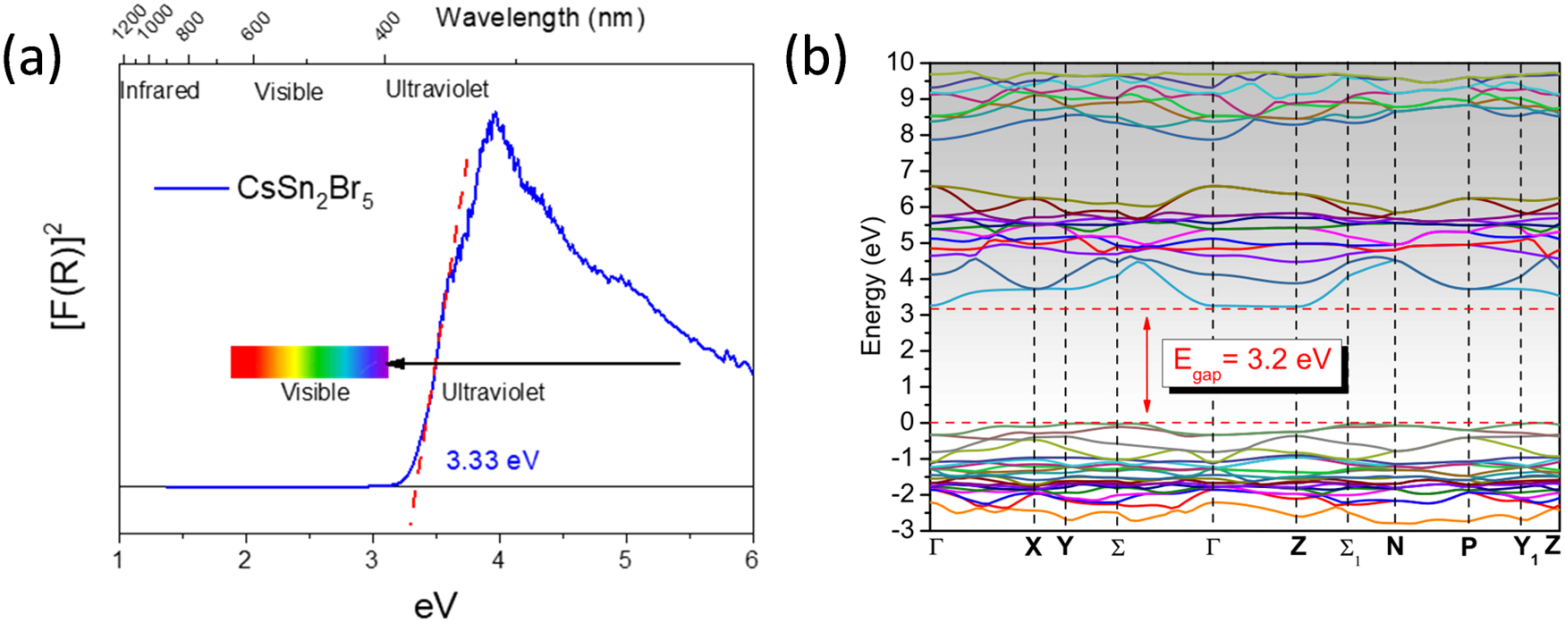
(a) Kubelka–Munk transformed diffuse reflectance spectrum of CsSn_2_Br_5_ (b). Band structure calculated from DFT methods for CsSn_2_Br_5_ showing an indirect gap transition (Σ→Ζ).

Figure 9a displays the Kubelka–Munk function against energy plot, indicating that the absorption band falls within the ultraviolet range The band gap estimated by extrapolating the linear region to the abscissa is ∼3.3 eV. This value is significantly higher than that of the 3D phase CsSnBr_3_ (1.8 eV),^53^ but it aligns with that reported for the 0D Cs_4_SnBr_6_ (3.34 eV).^54^ A similar trend is observed when comparing 0D, 2D, and 3D phases for the lead family (Cs/Pb/Br).^49^ Other 2D phases such as RbSn_2_Br_5_ (3.08 eV)^46^ and CsPb_2_Br_5_ (3.35 eV)^55^ exhibit band-gap values similar to those obtained for the present phase. The nature of the optical gap transition was found to be indirect (Σ→Ζ), as demonstrated by band structure in Figure 9b. The calculated transition (3.2 eV) is in excellent agreement with the experimental value from optical measurements (∼3.3 eV). Partial density of states can also be consulted in Figure S4.

### 4.7 Thermoelectric Performance

**Figure 10:**
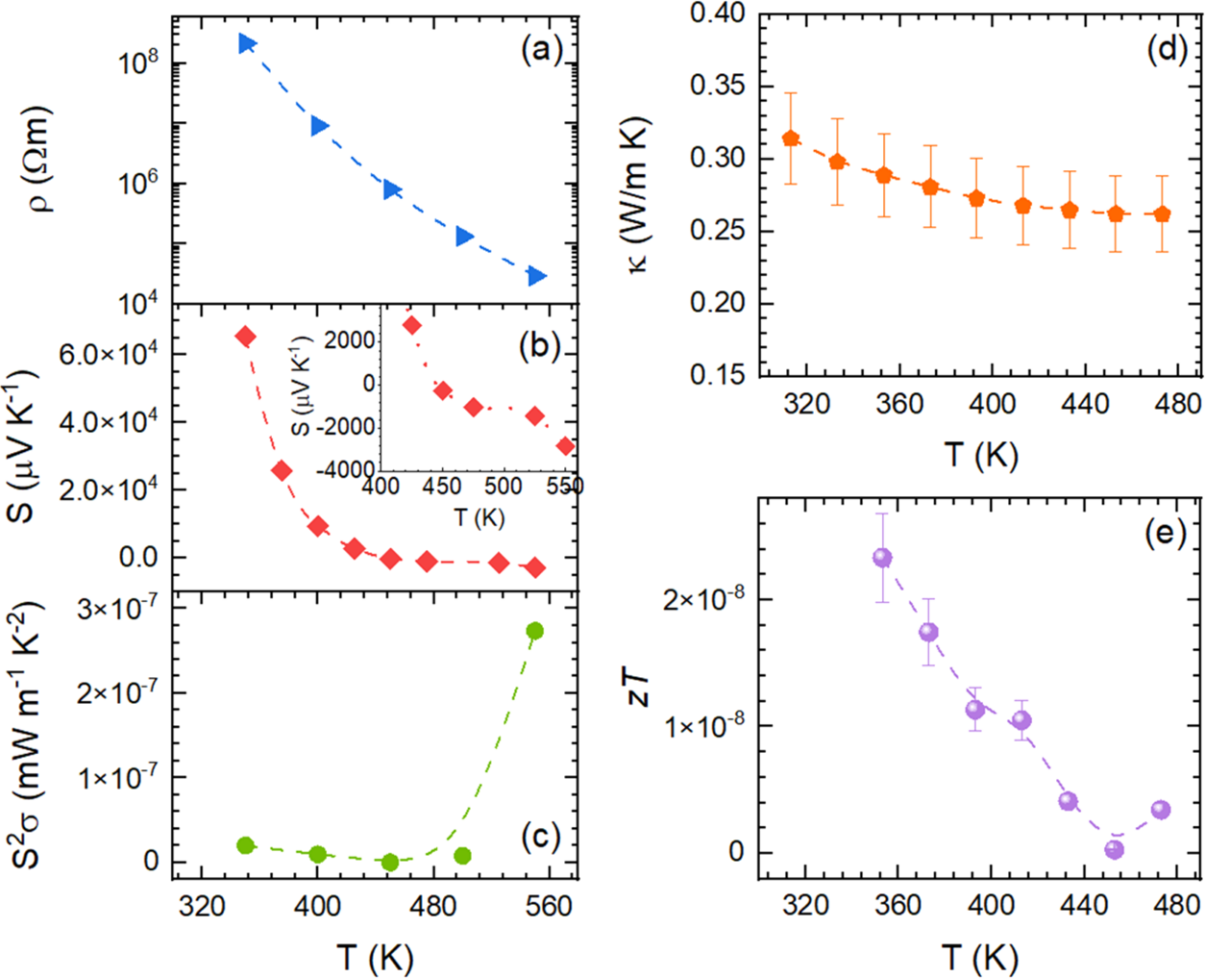
Electrical resistivity ρ (a), Seebeck coefficient *S* (b), power factor *S*^2^σ (c), thermal conductivity κ (d), and thermoelectric figure of merit *ZT* (e) for the CsSn_2_Br_5_ sample.

The transport properties of these 2D halide perovskites have been scarcely investigated, as previous reports are mostly focused on optoelectronic functionalization.^20^ In Figure 10, the temperature dependence of the thermoelectric transport properties of CsSn_2_Br_5_ is displayed. In agreement with the wide gap and intrinsic nature of this compound, the electrical resistivity (ρ) shows an exponential decrease from 2 × 10^8^ Ω m at 350 K down to 3 × 10^4^ Ω m at 550 K (see Figure 10a). These values are 2 orders of magnitude more resistive than the RbSn_2_Br_5_ derivative,^46^ despite its band gap of 3.3 eV^20^ compared to that of its Rb counterpart of 3.08 eV.^46^ Assuming a resistivity limited by charge carrier density and thermal activation, an Arrhenius energy of *E*_b_ = 0.64 eV is determined, which is close to that calculated for RbSn_2_Br_5_.

The Seebeck coefficient (*S*) in Figure 10b displays extremely large absolute values that resulted from the low carrier concentration in the sample, similarly to the Rb derivative, and much larger than those described in CsSnBr_3_.^31^ The Seebeck coefficient shows a decreasing trend along with increasing thermal activation of charge carriers from 6 × 10^4^ μV K^–1^ at 350 K down to negative values of −3 × 10^3^ μV K^–1^ at 550 K, as a steeper evolution with temperature than the Rb derivative. This effect could be anticipated by considering the Pisarenko relation, where, for a given effective mass, the Seebeck coefficient becomes smaller (in absolute value) as carrier density increases. Furthermore, the p–n crossover suggests a change from holes to electrons as the main contribution to the Seebeck coefficient and agrees well with a smaller band gap. The power factor (Figure 10c) is rather small at ∼10^–7^ mW m^–1^ K^–2^ in the whole temperature range as a consequence of the high resistivity, being much lower than that required for thermoelectric applications^23,25,56^ and in a similar range as for RbSn_2_Br_5_ and RbPb_2_Br_5_.^46,50^

The total thermal conductivity (κ) evolution with temperature is represented in Figure 10d, showing values between 0.32 and 0.25 W m^–1^ K^–1^. Due to the high resistivity, the electronic contribution is negligible and it completely represents the lattice contribution. These values are below those of other halide perovskites (0.8–0.3 W m^–1^ K^–1^) but slightly above those reported for RbSn_2_Br_5_ and RbPb_2_Br_5_.^46,50^ Most likely, this ultralow thermal conductivity is a consequence of weak bonding interactions within the lattice, as described by the Debye temperatures.

The combination of these properties results in *ZT* values, defined as *ZT* = *S*^2^σ*T*/κ, of ∼10^–7^ in this temperature range (see Figure 10e), which are rather low compared to that of materials for thermoelectric applications. Nevertheless, they are still in a similar range as the perovskite materials mentioned throughout the discussion, such as RbSn_2_Br_5_, RbPb_2_Br_5_,^46,50^ and MAPbI_3_.^33^

## 5 Conclusions

The CsSn_2_Br_5_ halide has been successfully synthesized as a well-crystallized powder using mechanochemical methods under an inert atmosphere. The crystal structure evolution was investigated by combining high angular resolution SXRD and NPD data. The 2D framework consists of layers of corner-sharing [SnBr_4_] polyhedra. X–N techniques permitted the location of the lone electron pairs of Sn^2+^, occupying an empty space opposite to the four Sn–Br chemical bonds of the pyramid. Cs^+^ cations are interleaved with the [Sn_2_Br_5_]^−^ layers along the *c*-axis. The analysis of the displacement factors within the Debye model revealed a distinct relative stiffness of Cs–Br versus Sn–Br pair bonds, and the topochemical maps indicated that the electron density is more concentrated along the Sn–Br pair bond than the Cs–Br pair, suggesting a more covalent character for the former one. The material exhibits thermoelectric properties comparable to those of other tin and lead halides, including a large Seebeck coefficient and low thermal conductivity, but with a very high electrical resistivity that results in a negligible thermoelectric figure of merit (∼10^–7^). The optical band gap of the ball-milled specimen was found to be 3.3 eV, being an indirect gap transition as calculated from the *B3LYP*-DFT method (∼3.2 eV).

## Supporting Information Available

SEM images and EDX spectra; evolution of the lattice constants with temperature; and partial density of states (PDOS) for CsSn_2_Br_5_ (PDF)
