# Supplementary material for: Unveiling the Structural Properties, Optical Behavior, and Thermoelectric Performance of 2D CsSn2Br5 Halide Obtained by Mechanochemistry
Source: Inorg Chem. 2024 Jun 26;63(27):12641–50. doi: 10.1021/acs.inorgchem.4c01861 (PMC11234366; doi:10.1021/acs.inorgchem.4c01861)
Supplement: Supplementary file 1 — ic4c01861_si_001.pdf [file ic4c01861_si_001.pdf]

SUPPLEMENTARY INFORMATION

Unveiling the structural properties, optical behavior, and thermoelectric performance of 2D $CsSn_2Br_5$ halide obtained by mechanochemistry

Authors:

Carlos Alberto López[1,2], Carmen Abia[1,3], Javier Gainza[4], João Elias Rodrigues[5], Brenda Martinelli[6], Federico Serrano-Sánchez[1], Romualdo Santos Silva Jr.[1], Mateus M. Ferrer[6], Oscar J. Dura[7], José Luis Martínez[1], María Teresa Fernández-Díaz[3], and José Antonio Alonso[1]*

Affiliations:

[1]*Instituto de Ciencia de Materiales de Madrid, CSIC, Cantoblanco, 28049, Madrid, Spain.*

[2]*INTEQUI, (UNSL-CONICET) and Facultad de Química, Bioquímica y Farmacia, UNSL, Almirante Brown 1455, 5700, San Luis, Argentina.*

[3]*Institut Laue Langevin. 38042 Grenoble Cedex, France.*

[4]*European Synchrotron Radiation Facility (ESRF), 71 Avenue des Martyrs, 38000 Grenoble, France.*

[5]*CELLS–ALBA synchrotron Light Facility, Cerdanyola del Valles, Barcelona, E-08290, Spain.*

[6]*CCAF, PPGCEM/CDTec, Federal University of Pelotas, 96010-610 Pelotas, Rio Grande do Sul, Brazil.*

[7]*Departamento de Física Aplicada, Universidad de Castilla-La Mancha, Ciudad Real, E-13071, Spain.*

**Corresponding author: ja.alonso@icmm.csic.es*

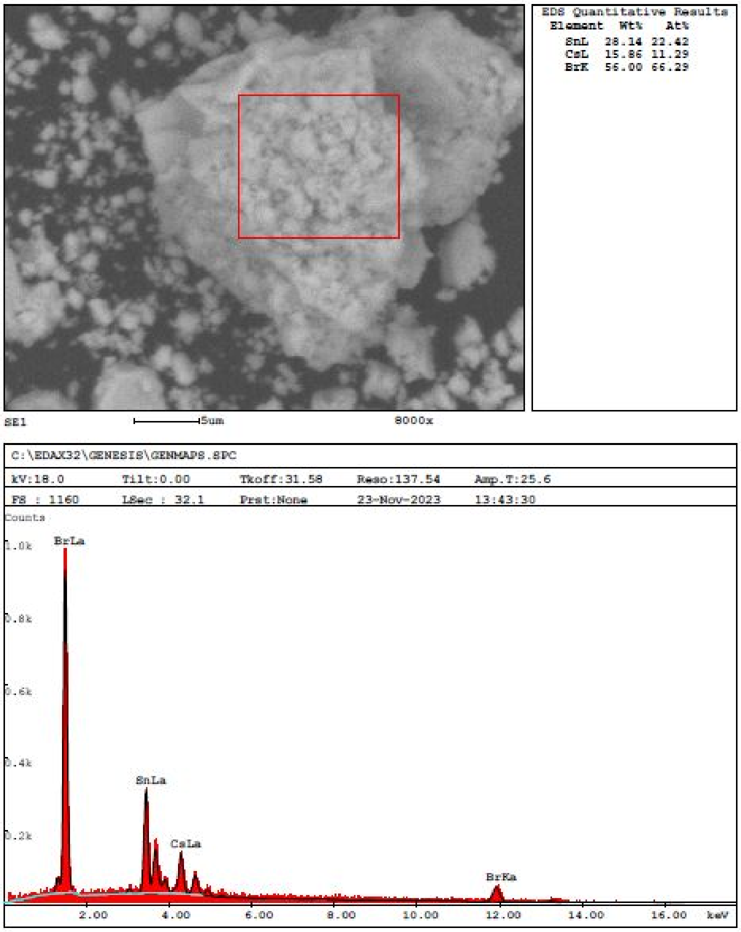

Fig. S1: Upper panel: SEM image where the EDX spectrum was collected, and relative contents of Cs, Sn and Br, very close to the expected composition $CsSn_2Br_5$. Lower panel: typical EDX spectrum.

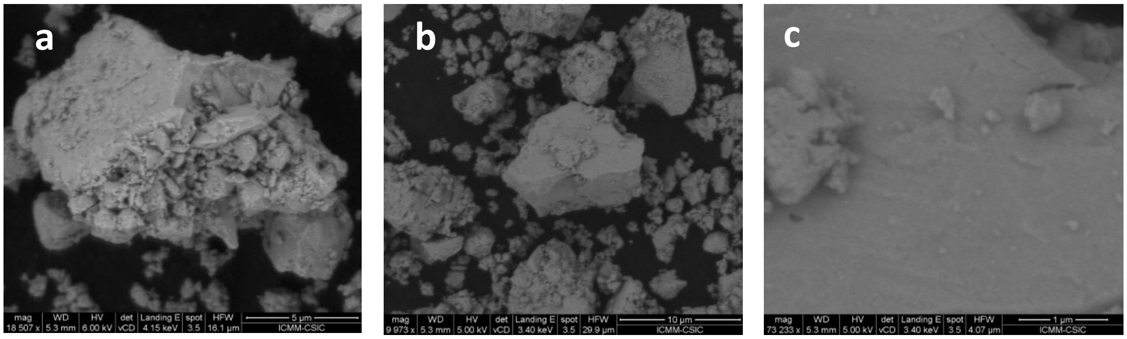

Fig. S2: FE-SEM images with 18,507× (a), 9973× (b), and 73,233× (c) magnification.

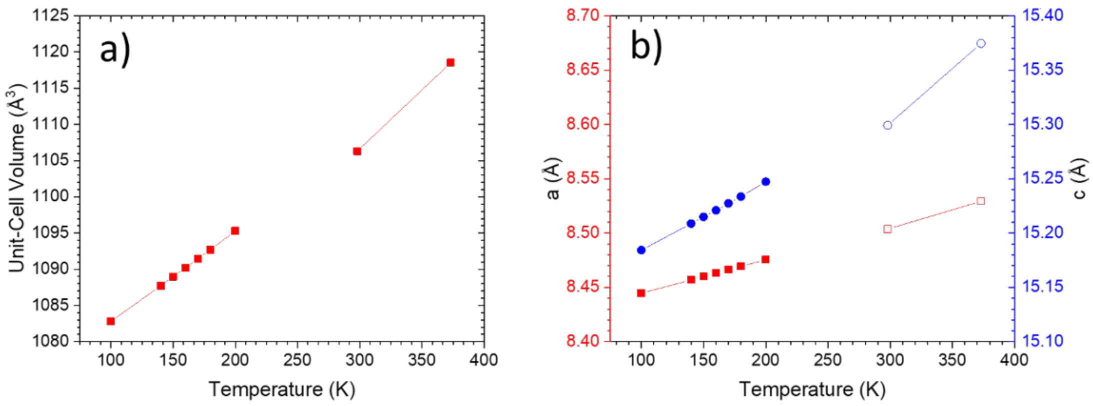

Fig. S3: Plots of the evolution of the lattice constants *a*, *c*, and *V* of $CsSn_2Br_5$ with temperature as obtained from SXRD data.

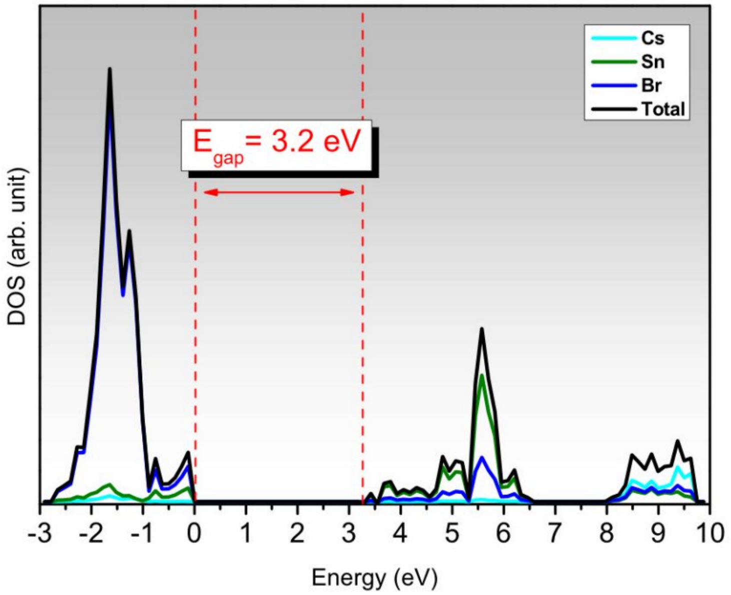

Fig. S4: Partial density of states (PDOS) for $CsSn_2Br_5$ halide, showing a bandgap transition of 3.2 eV (B3LYP-DFT method).
